# Supplementary material for: Ab initio quantum transport in AB-stacked bilayer penta-silicene using atomic orbitals
Source: RSC Adv. 2018 Oct 3;8(59):34041–6. doi: 10.1039/c8ra05652h (PMC9086686; doi:10.1039/c8ra05652h)
Supplement: RA-008-C8RA05652H-s001 [file RA-008-C8RA05652H-s001.pdf]

Electronic Supplementary Material (ESI) for RSC Advances.
This journal is © The Royal Society of Chemistry 2018

Supplementary Information for: 'Ab initio quantum transport in AB-stacked bilayer penta-silicene using atomic orbitals'

Eleni Chatzikyriakou[1]*, Padeleimon Karafiloglou,[2], Joseph Kioseoglou[1]

August 30, 2018

1. Department of Solid State Physics, Aristotle University of Thessaloniki, Thessaloniki, 54124, Greece.

2. Department of Chemistry, Aristotle University of Thessaloniki, Thessaloniki, 54124, Greece.

1 Wannier functions

The convergence of the Wannier functions was checked both from their imaginary parts and from plotting the bandstructure. Figure 1 shows a comparison of the bandstructure in a portion of the reciprocal space from Wannier and DFT results. Higher in the conduction band, the highly entangled eigenstates reproduce poorly, however, for transport simulations, we are mainly interested in the states around the bandgap. Even in this case, where most of the orbitals acquire a less localized sp nature, the system was efficiently simulated.

*E-mail: elchatz@auth.gr

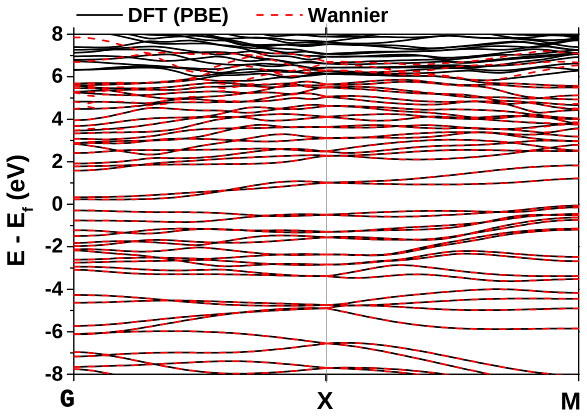

Figure 1: Comparison between the band structure results from Wannier functions and DFT calculations with PBE exchange correlation functional.

2 Band structure from Tight Binding model

The band structure was checked for its correctness in the Kwant code [1]. A 1x1 system with three nearest neighbours was used in Kwant with 3D translational symmetry, where the dispersion relations were derived for the high symmetry points in the Brillouin zone (fig. 2).

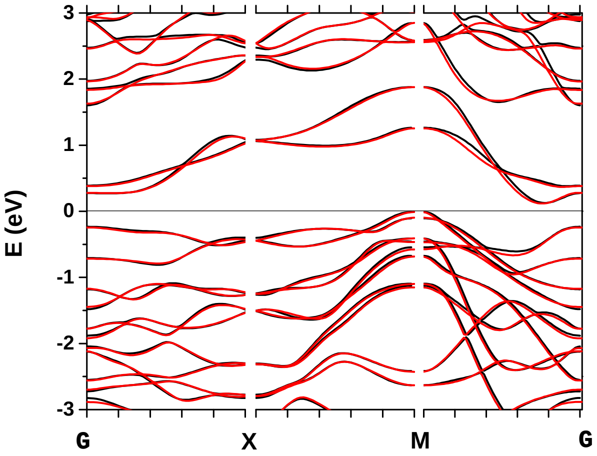

Figure 2: Dispersion relations in Kwant (black lines) and band structure from DFT code with PBE functional (red lines).

3 Quantum conductance

The accuracy of quantum transport simulation increases with the order of the nearest neighbor (nn) hoppings. Conductance calculation results with different nn hoppings are shown in Figure 3 with 0 being nearest neighbour, 1 next-nearest neighbour and so on. The conductance is calculated from [1]

$$G_{ab} = \frac{2e^2}{h} MT = \frac{2e^2}{h} \sum_{n \in a, m \in b} |S_{nm}|^2 \quad (1)$$

We observe differences in the conductance of the medium when allowing hoppings to more distant neighbours which is attributed to the differences in the scattering probability that occurs due to the changes in the eigenenergies of the Hamiltonian in the leads. Increasing the width of the wire amounts to an increase in the magnitude of its conductance in discrete steps, according to the Landauer formula, while with a sufficient increase in the number of atoms in the system, the Ohmic behaviour is restored,

$$G_{ab} = \sigma \frac{W}{L} \quad (2)$$

where σ is the conductivity of the material, W is the width and L is the length of the semiconductor region.

For calculating current-voltage characteristics of the wire, summing over all propagating modes close to the fermi level would be needed. In this case, finite temperature effects and any non-linearities will properly need to be addressed.

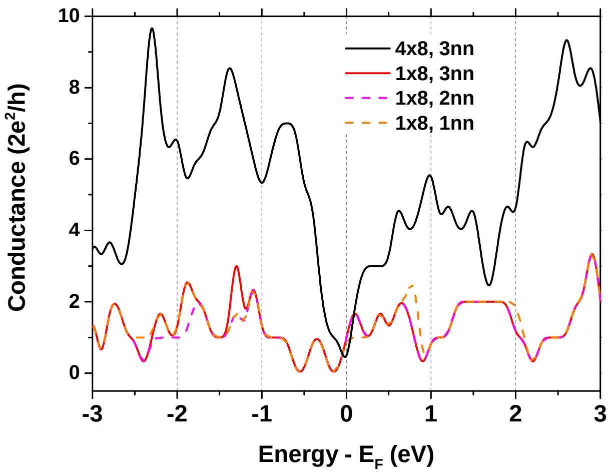

Figure 3: Conductance results for a scattering region of 1x8 and 4x8 W/L and nearest neighbour hoppings of order (nn) as indicated - 1 being next nearest neighbours. The energy step is 0.1 eV. The length extends in the k_x direction.

References

[1] Christoph W. Groth, Michael Wimmer, Anton R. Akhmerov, and Xavier Waintal. Kwant: a software package for quantum transport. *New Journal of Physics*, 16(6):063065, jun 2014. doi: 10.1088/1367-2630/16/6/063065.
